# Efficient Formaldehyde Gas Sensing Performance via Promotion of Oxygen Vacancy on In-Doped LaFeO_3_ Nanofibers

**DOI:** 10.3390/nano14191595

**Published:** 2024-10-02

**Authors:** Lei Zhu, Jiaxin Zhang, Jianan Wang, Jianwei Liu, Wei Zhao, Wei Yan

**Affiliations:** 1 Xi’an Key Laboratory of Solid Waste Resource Regeneration and Recycling, State Key Laboratory of Multiphase Flow Engineering, School of Energy and Power Engineering, Xi’an Jiaotong University, Xi’an 710049, China; leizhu@xjtu.edu.cn (L.Z.);; 2 School of Physics and Electrical Engineering, Weinan Normal University, Chaoyang Street, Weinan 714099, China; 3 School of Chemistry and Chemical Engineering, Xi’an University of Science & Technology, Xi’an 710054, China

**Keywords:** LaFeO_3_, oxygen vacancy, doping, formaldehyde detection, nanofibers

## Abstract

Perovskite oxide LaFeO_3_(LFO) emerges as a potential candidate for formaldehyde (HCHO) detection due to its exceptional electrical conductivity and abundant active metal sites. However, the sensitivity of the LFO sensor needs to be further enhanced. Herein, a series of La_x_In_1-x_FeO_3_ (x = 1.0, 0.9, 0.8, and 0.7) nanofibers (L_x_In_1-x_FO NFs) with different ratios of La/In were obtained via the electrospinning method followed by a calcination process. Among all these L_x_In_1-x_FO NFs sensors, the sensor based on the L_0.8_In_0.2_FO NFs possessed the maximum response value of 18.8 to 100 ppm HCHO at the operating temperature of 180 °C, which was 4.47 times higher than that based on pristine LFO NFs (4.2). Furthermore, the L_0.8_In_0.2_FO NFs sensor also exhibited a rapid response/recovery time (2 s/22 s), exceptional repeatability, and long-term stability. This excellent gas sensing performance of the L_0.8_In_0.2_FO NFs can be attributed to the large number of oxygen vacancies induced by the replacement of the A-site La^3+^ by In^3+^, the large specific surface area, and the porous structure. This research presents an approach to enhance the HCHO gas sensing capabilities by adjusting the introduced oxygen vacancies through the doping of A-sites in perovskite oxides.

## 1. Introduction

Formaldehyde (HCHO) has garnered considerable public concern owing to its harmful effects on human health [1,2]. Exposure to elevated levels of HCHO is associated with a variety of negative health effects [3]. Short-term exposure can cause symptoms such as eye, nose, and throat irritation, along with wheezing and skin rashes. Prolonged HCHO exposure has been linked to more severe health hazards, including respiratory problems, nasopharyngeal cancer and leukemia [4]. The World Health Organization (WHO) has established a guideline value of 81 ppb for the exposure threshold of HCHO [5,6]. Given the potential health implications, the capability to detect HCHO in real time is essential for the protection of human health.

Gas sensors based on metal oxide semiconductors (MOSs) have attracted more attention in the field of gas detection [7,8,9]. The p-type semiconductor LaFeO_3_, featuring the ABO_3_ perovskite structure, presents greater potential for achieving high sensitivity and selectivity in gas detection due to its high electrical conductivity, abundant active metal sites, and excellent oxidation–reduction characteristics [10,11]. For example, Zhang et al. [12] synthesized porous LaFeO_3_ particles for the HCHO gas sensor by the sol-gel technique. The presented sensor exhibited a high level of response and excellent selectivity toward HCHO. Sun et al. [13] reported that the sensor based on LaFeO_3_ nanofibers/Ti_3_C_2_T_x_ MXene sensor achieved superior sensitivity and enhanced stability for HCHO gas detection due to the p-n heterojunction of the LaFeO_3_/Ti_3_C_2_T_x_ composite. Despite considerable advancements, the inherent sensitivity of p-type LaFeO_3_ (LFO) sensors remains insufficient for commercial application.

The engineering of surface defects, such as oxygen vacancies, is essential for boosting the sensitivity of gas sensors, which can offer additional active sites for gas adsorption, catalyze interfacial reactions, and modulate the electronic structure of the sensing materials [14,15]. Doping at the A- and B-sites of perovskite has been validated as a potent strategy for the creation of oxygen vacancies by substituting ions within the original compound [16,17,18]. For instance, Sun et al. [19] developed a calcium-doped perovskite, and their finding indicated that more oxygen vacancies were generated due to the Ca^2+^ ion doping. Duan et al. [20] reported that the improvement of the CO_2_ gas sensing capabilities of LaFeO_3_ by introducing Co doping facilitated the formation of oxygen vacancies. Indium ion (In^3+^) possesses a +3 oxidation state and its ionic radius is similar to La, which is suitable as a dopant for improving the sensitivity of the LaFeO_3_ sensors.

In addition, one-dimensional (1-D) nanofibers exhibit enhanced electron migration in the radial direction and a high surface area-to-volume ratio, which significantly contribute to the significant change in resistance [21]. Consequently, this leads to enhancement of the sensitivity of the gas sensor. Compared with hydrothermal and template methods, electrospinning can provide a facile and versatile route to prepare 1-D nanostructures [22]. By controlling the experimental conditions and post-processing methods of electrospinning, the morphology, structure, composition and even the macroscopic appearance of the fibers can be regulated.

Herein, La_x_In_1-x_FeO_3_ nanofibers (L_x_In_1-x_FO NFs) with different ratios of La/In were synthesized using the electrospinning method followed by a calcination post-treatment. The gas sensing performance of the L_x_In_1-x_FO NFs sensors toward HCHO gas was investigated. Among these L_x_In_1-x_FO NFs sensors, the gas sensor based on L_0.8_In_0.2_FO NFs showed the highest sensitivity of 18.8 in response to 100 ppm HCHO at an operating temperature of 180 °C, surpassing the sensitivity of the pristine LFO NFs (4.2). In addition, the L_0.8_In_0.2_FO NFs sensor exhibited rapid response/recovery speed (2 s/22 s) and excellent long-term stability to HCHO. Further analysis indicated that the improved HCHO sensing capabilities of L_0.8_In_0.2_FO NFs could be ascribed to the substantial generation of oxygen vacancies from the In^3+^ quantitative substitution for La^3+^ in the A-site. Additionally, the increased surface area and the porous structure of the L_0.8_In_0.2_FO NFs also contributed to this enhanced HCHO gas sensing performance.

## 2. Experimental Section

### 2.1. Synthesis of La_x_In_1-x_FeO_3_ Nanofibers (L_x_In_1-x_FO NFs)

**Figure 1:**
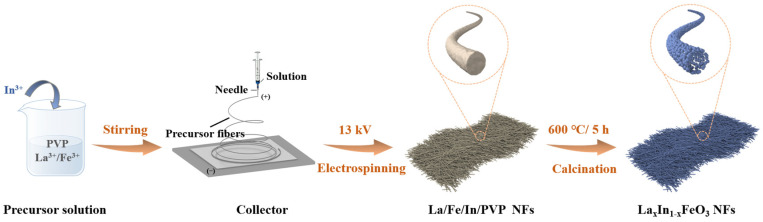
Schematic illustration of the synthetic process for the La_x_In_1x_FeO_3_ NFs.

The L_x_In_1-x_FO NFs (x = 1.0, 0.9, 0.8, and 0.7) were fabricated utilizing the electrospinning methodology followed by a subsequent annealing process. Here, 0.808 g of Fe (NO_3_)_3_·9H_2_O, and certain La/In molar ratios of La(NO_3_)_2_⋅6H_2_O and In (NO_3_)_3_⋅4.5H_2_O were added into in a mixture comprising 5 mL of ethanol and 10 mL of N,N-dimethylformamide and then thoroughly mixed to achieve a homogeneous solution. Then, 1 g of PVP was added to the above mixture and the solution was stirred for 6 h. The precursor fibers were then subjected to a subsequent calcination stage. This involved heating the fibers to 600 °C for 5 h with a controlled heating rate of 10 °C· min^−1^ in air to ultimately produce the L_x_In_1-x_FO nanofibers. In this process, the molar of La/In ratios were 1:0, 9:1, 8:2, 7:3, corresponding to the LFO, L_0.9_In_0.1_FO, L_0.8_In_0.2_FO, and L_0.7_In_0.3_FO samples, respectively. The preparation process for the L_x_In_1-x_FO nanofibers is shown in Figure 1.

### 2.2. Characterizations

The microscopic morphologies of the nanofibers were examined using a scanning electron microscope (SEM, GeminiSEM 500, Singapore) and transmission electron microscopy (TEM, Talos-F200X, Waltham, MA, USA). The patterns of X-ray diffraction (XRD) were obtained using an XRD-6100 spectrometer (Kyoto, Japan), which employs a Cu Kα radiation source for the analysis. The Fourier transform infrared spectroscopy (FT-IR) analysis was performed using a Bruker Tensor 37 spectrometer. The Raman scattering analysis was conducted using an HR800 Raman spectrometer from France, with excitation provided by a 633 nm laser light source. The specific surface area and pore size distribution of the samples were measured via N_2_ adsorption/desorption isotherms using an SSA-4300 (Shanghai, China) instrument. The X-ray photoelectron spectroscopy (XPS) data were acquired using an AXIS ULtrablD instrument from the Wokingham, UK. The UV-visible diffuse reflectance spectra were determined on a PE Lambda950 (Suzhou, China) spectrophotometer. The photoluminescence (PL) spectra of the samples were recorded using a steady state and lifetime fluorescence spectrometer (FLS1000, Livingston, UK).

### 2.3. Gas Sensing Measurements

The assessment of the gas sensing performance was conducted utilizing the WS-30A system (Wei Sheng Electronics Co., Ltd., Guangzhou, China). The sensing cycle involved three sequential stages: (I) exposure to ambient air; (II) exposure to tested gases to trigger a response signal; and (III) subsequent exposure to ambient air to facilitate a return to the baseline condition. A detailed description of the gas sensor fabrication and sensing performance tests is presented in Supplementary Figure S1 and Text S1. The gas sensor’s response was defined as: S = R_g_/R_a_, wherein R_g_ and R_a_ denote the resistive values in the presence of the target gas and ambient air, respectively. The response time and recovery time are the times needed for the sensor to reach 90% of its maximum resistance value and to recover to 90% of its initial resistance value, respectively.

## 3. Results and Discussion

### 3.1. Microstructures and Composition

**Figure 2:**
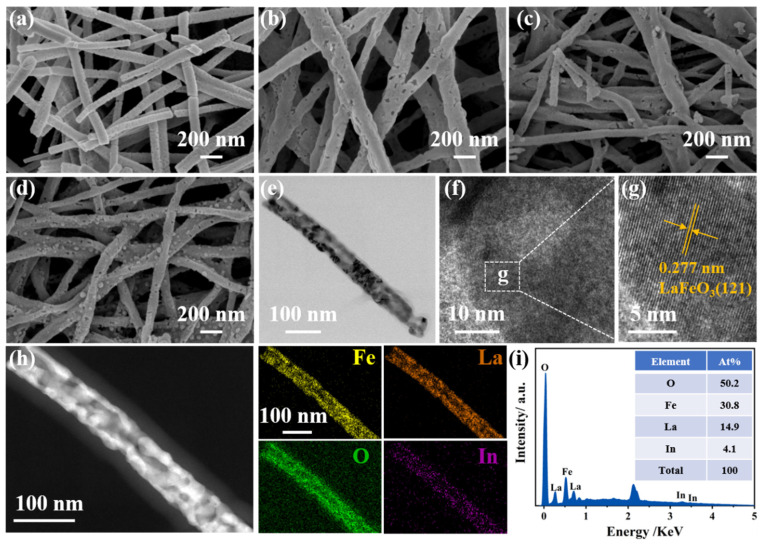
SEM images of the (**a**) LFO, (**b**) L_0.9_In_0.1_FO, (**c**) L_0.8_In_0.2_FO, and (**d**) L_0.7_In_0.3_FO samples; (**e**) TEM and (**f**,**g**) the corresponding HRTEM image; and (**h**) EDS element mapping and (**i**) the EDS spectrum of L_0.8_In_0.2_FO NFs.

The SEM images of the LFO, L_0.9_In_0.1_FO, L_0.8_In_0.2_FO, and L_0.7_In_0.3_FO NFs are presented in Figure 2a–d, respectively. All the L_x_In_1-x_FO samples possess uniform nanofiber morphology consisting of numerous uniformly sized nanoparticles. In addition, with the increasing indium doping, many pores appeared on the surface of the L_0.9_In_0.1_FO, L_0.8_In_0.2_FO, and L_0.7_In_0.3_FO NFs. In addition, the emergence of nanoparticles on the surface of the L_0.7_In_0.3_FO NFs is observed, likely attributable to the excessive doping of indium ions within the crystal structure (Figure 2d). The TEM image in Figure 2e demonstrates that L_0.8_In_0.2_FO has a hollow nanofiber structure with a fiber diameter of about 55 nm. Figure 2f,g show the HRTEM images of L_0.8_In_0.2_FO NFs, where the lattice fringes with d-spacing of 0.227 nm are indicative of the (121) plane of the LaFeO_3_ perovskite oxide structure [23,24]. Figure 2h illustrates the EDS elemental mappings, which indicate an even distribution of Fe, La, O, and In elements throughout the L_0.8_In_0.2_FO NFs. This uniform distribution confirms the successful incorporation of indium into the LaFeO_3_ perovskite [16,18]. The EDS spectrum depicted in Figure 2i verifies the atom ratios of the constituent elements of the L_0.8_In_0.2_FO NFs. The La/In molar ratio of L_0.8_In_0.2_FO NFs is calculated as 3.6, which is slightly lower than the expected set molar ratio of 4.0.

**Figure 3:**
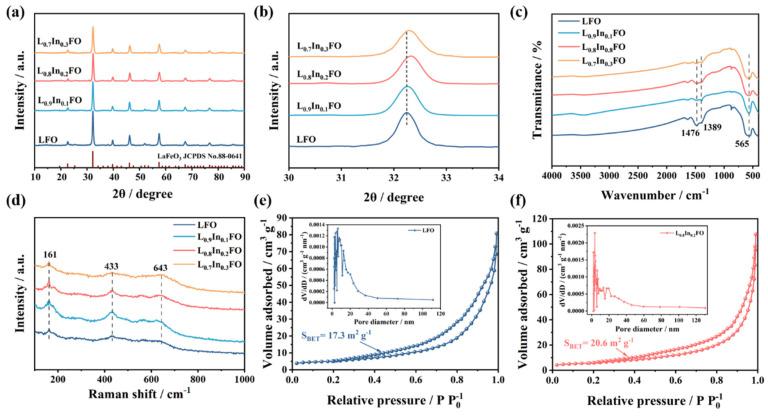
(**a**) Full angle range of XRD patterns and (**b**) enlarged XRD peaks of the L_x_In_1-x_FO samples; (**c**) FT-IR spectra and (**d**) Raman spectra of four L_x_In_1-x_FO samples; and N_2_ adsorption–desorption isotherms and pore size distributions of the (**e**) LFO and (**f**) L_0.8_In_0.2_FO NFs.

The XRD patterns of the different L_x_In_1-x_FO (x = 1.0, 0.9, 0.8, and 0.7) samples are displayed in Figure 3a. The XRD data reveal that the diffraction peaks for all the L_x_In_1-x_FO are consistent with the standard JCPDS card (88-0641) of LaFeO_3_, confirming the characteristic orthorhombic perovskite structure of the synthesized materials [20]. The magnified view of the XRD diffraction peaks (Figure 3b) reveals a shift toward higher angles with the increase of the indium content, which can be ascribed to the partial substitution of La^3+^ (ionic radius 1.03 Å) with smaller In^3+^ ions (ionic radius 0.80 Å) in the A-site [25,26]. This result demonstrates that the In^3+^ dopant has been incorporated into the lattice of LaFeO_3_.

The FT-IR analysis was conducted to provide an understanding of the chemical composition and the surface functional groups present in the L_x_In_1-x_FO NFs (Figure 3c). All these L_x_In_1-x_FO samples exhibit absorption bands at 565 cm⁻^1^, indicative of Fe–O stretching vibrations [27,28]. Furthermore, the absorption bands observed at 1389 cm^−1^ are associated with the CO_3_^2−^ group due to exposure to the ambient atmosphere [16]. The absorption bands at 1476 cm^−1^ are indicative of the stretching vibrations of the -OH groups from water molecules that are adsorbed onto the surface of the samples [29].

To gain a deeper comprehension of the local chemical structure, the Raman spectrum of the LFO, L_0.9_In_0.1_FO, L_0.8_In_0.2_FO, and L_0.7_In_0.3_FO NFs were performed, as depicted in Figure 3d. The three main bands in the Raman spectra of the samples, located at around 161, 433, and 643 cm^−1^, are indicative of the A_g_ vibration mode associated with the La cation, B_3g_ bending vibration mode (B) of the FeO_6_ octahedral, and the symmetric stretching vibration of the octahedral lattice oxygen, respectively [30,31]. Nevertheless, in comparison with the LFO sample, the intensity of the Raman peaks is significantly enhanced with the increased amount of indium doping due to the induced lattice distortion from the replacement of the La^3+^ with In^3+^ ions within the LFO lattice structure [32].

The specific surface area and pore characteristics of all the L_x_In_1-x_FO samples were evaluated through nitrogen adsorption–desorption measurements, as depicted in Figure 3e,f and Figure S2. The nitrogen adsorption–desorption isotherms of all four samples contribute to a characteristic type IV curve accompanied by an H3 hysteretic loop, indicating the presence of numerous mesopores structures. The specific surface areas of the LFO L_0.9_In_0.1_FO, L_0.8_In_0.2_FO, and L_0.7_In_0.3_FO NFs are 17.3, 19.5, 20.6, and 21.7 m^2^ g^−1^, respectively. The increased surface area of the L_0.8_In_0.2_FO NFs indicates an enhanced capacity for gas adsorption [33]. Meanwhile, the calculated pore size of the L_0.8_In_0.2_FO NFs is mainly distributed about 3.5 nm, which is smaller than that of the LFO NFs (6.1 nm). In addition, the L_0.8_In_0.2_FO NFs exhibit an increased presence of a mesoporous structure characterized by pore sizes of less than 5 nm [29]. The increased specific surface area and porosity of the L_0.8_In_0.2_FO NFs are conducive to amplifying the sensor’s sensitivity and response speed.

**Figure 4:**
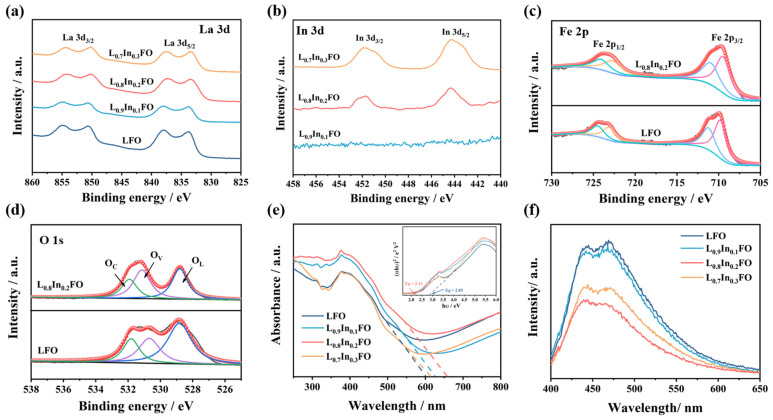
XPS spectra of the (**a**) La 3d of four L_x_In_1-x_FO samples, (**b**) In 3d of the L_0.9_In_0.1_FO, L_0.8_In_0.2_FO, and L_0.7_In_0.3_FO NFs, (**c**) O 1s, and (**d**) Fe 2p of the LFO and L_0.8_In_0.2_FO NFs; and (**e**) UV–vis diffuse reflectance spectra and (**f**) PL measurement of four L_x_In_1-x_FO NFs.

The XPS analysis was conducted to further explore the surface chemical binding states of the L_x_In_1-x_FO samples. The XPS survey spectrum verifies the existence of La, Fe, and O elements within the LFO NFs (Figure S3). In contrast to the LFO and L_0.9_In_0.1_FO NFs, the survey spectra of the L_0.8_In_0.2_FO and L_0.7_In_0.3_FO NFs exhibit additional In 3d peaks. As displayed in Figure 4a, the peaks observed at 833.3 and 850.1 eV corresponding to the La 3d_5/2_ and La 3d_3/2_, respectively, can be assigned to the La^3+^ oxidation state [34]. As for the In 3d spectra (Figure 4b), the peaks corresponding to the In 3d_5/2_ and In 3d_3/2_ of In^3+^ at 444.3 and 451.8 eV are observed in the L_0.8_In_0.2_FO and L_0.7_In_0.3_FO NFs, proving the introduction of indium into the LFO perovskite structure [20,35]. From the Fe 2p spectra of the LFO and L_0.8_In_0.2_FO NFs in Figure 4c, the double peaks at 709.5 and 722.8 eV can be attributed to the 2p_3/2_ and 2p_1/2_ states of Fe^3+^, respectively, while the peaks at 711.2 and 724.3 eV are assigned to the 2p_3/2_ and 2p_1/2_ states of Fe^4+^, respectively [36]. In the high-resolution O1 s spectra, the fitting peaks are deconvoluted into lattice oxygen (O_L_), oxygen vacancies (O_V_), and chemisorbed oxygen (O_C_), located at 528.9, 531.5, and 532.3 eV, respectively (Figure 4d and Figure S4). The proportion of the three oxygen content types for the L_x_In_1-x_FO samples is presented in Table S1. The L_0.8_In_0.2_FO NFs exhibit the highest content of O_V_ (38.9%) compared to the other L_x_In_1-x_FO samples. The elevated level of O_V_ can be ascribed to the appropriate replacement of La^3+^ by In^3+^, which leads to the generation of oxygen vacancy defects [37].

To assess the influence of varying molar ratios of In^3+^ on the bandgap, the UV–vis diffuse reflectance spectra of all the L_x_In_1-x_FO were determined, as depicted in Figure 3b. The optical absorption edge of LFO is located at 601 nm. In contrast, the maximum absorption edges of the L_x_In_1-x_FO (x = 0.9, 0.8, and 0.7) samples exhibit an apparent red shift with the increased In^3+^ concentration. This phenomenon suggests that the doping of In^3+^ significantly modulates the electronic energy levels of the samples [15]. In addition, the band gap of all the samples was calculated using the Tauc’s plot employing the Kubelka–Munk function with the equation as below:

αhν = A(hν − E_g_)^n^ (1)

where α is the absorption coefficient, h is Planck’s constant, ν is the frequency of vibration, A is a proportionality constant, and E_g_ is the band gap [38,39]. The electron transition factor n = 1/2 is for direct band gap calculations and n = 2 for the indirect band gap. The band gap value can be obtained from a plot of (αhν)^2^ vs. photon energy (hν) by the intercept of the tangent to the x-axis, as shown in the inset of Figure 4e [40]. The band gaps of the LFO, L_0.9_In_0.1_FO, L_0.8_In_0.2_FO, and L_0.7_In_0.3_FO NFs are 2.85, 2.23, 2.13, and 2.73 eV, respectively. This reveals that In^3+^ doping can effectively reduce the bandgap of the L_0.8_In_0.2_FO NFs, which can be attributed to the creation of oxygen vacancies by replacing La^3+^ with In^3+^ ions [27].

The photoluminescence (PL) spectrum analysis is another effective method for identifying the existence of different types of defect states in materials. Therefore, the room temperature PL emission spectra of all the L_x_In_1-x_FO samples with an excitation wavelength of 365 nm have been recorded (Figure 4f). The LFO NFs show the highest intensity in the PL spectra, indicative of the recombination of charge carriers [31]. Conversely, the PL emission intensities of the L_0.9_In_0.1_FO, L_0.8_In_0.2_FO and L_0.7_In_0.3_FO NFs remarkably decrease as the concentrations of oxygen vacancies rise, which is also consistent with the results of the XPS and UV-vis DRS measurements.

### 3.2. Gas Sensing Properties

**Figure 5:**
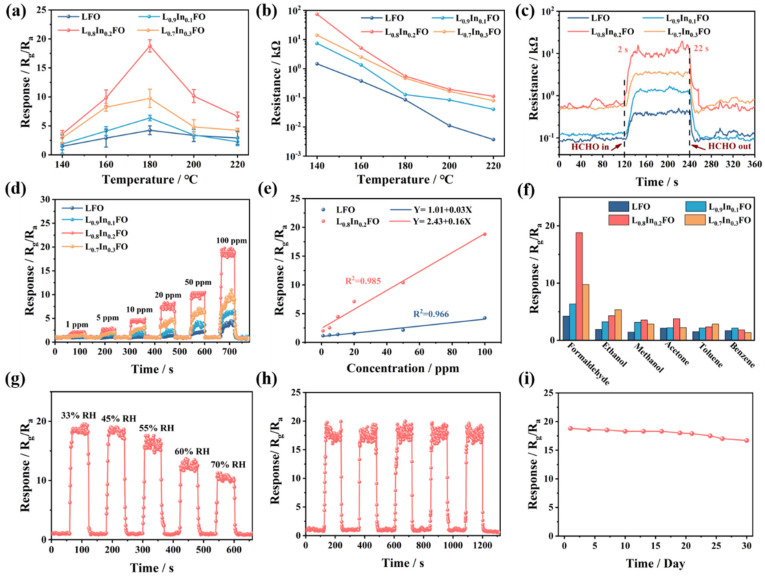
Sensing property measurements for the sensors based on the LFO, L_0.9_In_0.1_FO, L_0.8_In_0.2_FO, and L_0.7_In_0.3_FO NFs at 180 °C. (**a**) Response curves of the L_x_In_1-x_FO NFs to 100 ppm HCHO under different operating temperatures; (**b**) base resistance in air of the four sensors at different operating temperatures; (**c**) response and recovery characteristics of the sensors exposed to 100 ppm of HCHO; (**d**) responses curve of the sensors to 1–100 ppm of HCHO; (**e**) the linearity between the concentration of HCHO gas and the response value of the sensor; (**f**) selectivity of the four sensors to 100 ppm of various gases; (**g**) the responses of the L_0.8_In_0.2_FO sensor toward 100 ppm HCHO under different relative humidity conditions; (**h**) the repeatability of the L_0.8_In_0.2_FO sensor to HCHO (100 ppm); and (**i**) long-term stability curve of the L_0.8_In_0.2_FO sensor for 100 ppm of HCHO.

The optimal operating temperature is a critical parameter for the performance of gas sensors, significantly influencing the sensitivity and power consumption of the sensors in practical applications [41]. Figure 5a indicates the response of four sensors based on the LFO, L_0.9_In_0.1_FO, L_0.8_In_0.2_FO, and L_0.7_In_0.3_FO NFs toward 100 ppm HCHO under different operating temperatures. All the sensors demonstrate a volcano-shaped response trend as the operating temperature rises from 140 to 220 °C. At operating temperatures below 160 °C, the sensor responses are relatively weak due to the insufficient electron mobility of the sensing materials and an insufficient supply of the thermal energy needed to overcome the high reaction energy barrier. As the temperature increases, the response values of the four sensors gradually increase and reach a maximum in relation to 100 ppm HCHO at 180 °C, which are 4.2 (LFO), 6.35 (L_0.9_In_0.1_FO), 18.8 (L_0.8_In_0.2_FO), and 9.7 (L_0.7_In_0.3_FO), respectively. Among them, the L_0.8_In_0.2_FO NFs present the maximum response value of 18.8 toward HCHO, which is 4.47 times higher than that of the LFO NFs (4.2). When the temperature exceeds 180 °C, the response values decrease with the rising temperature. Therefore, the subsequent measurements of the gas sensing performance were carried out at the optimal operating temperature of 180 °C.

Figure 5b investigates the impact of various temperatures on the initial resistance values of the sensors. It shows that the resistance values of all the sensors decrease with the increased temperature, conforming to the characteristics of semiconductors [13]. Due to the reduced carrier concentration resulting from the incorporation of In^3+^ dopants, the resistance values in air of the L_0.9_In_0.1_FO, L_0.8_In_0.2_FO, and L_0.7_In_0.3_FO sensors are larger compared to the LFO NFs sensor at the same temperature. In addition, Figure 5c displays the dynamic response and recovery curves of the four sensors when exposed to 100 ppm HCHO at 180 °C. The sensors demonstrate the characteristic resistance change behavior of p-type oxide semiconductors. Upon exposure to HCHO, the resistance of the gas sensors increases rapidly and then stabilizes after a certain period. When the sensors return to the air environment, the resistance of the sensor recovers to its initial value. The response/recovery time of the L_0.8_In_0.2_FO sensor (2 s/22 s) is faster than that of pristine LFO (8 s/24 s). This improved performance can be ascribed to the larger surface area and porous structure of the L_0.8_In_0.2_FO NFs, facilitating the diffusion of the HCHO gas molecules throughout the sensing material and accelerating the sensing reaction [42].

Figure 5d illustrates the response curves of the four L_x_In_1-x_FO sensors toward HCHO gas in different concentration ranges. Specifically, the responses of all the sensors increase gradually with the increased HCHO concentration. The L_0.8_In_0.2_FO sensor is the most sensitive to HCHO at various concentrations among these sensors. Furthermore, Figure 5e demonstrates that the LFO and L_0.8_In_0.2_FO sensors have a good linear relationship between the response values and the HCHO concentration, manifesting potential for quantitative HCHO gas detection. Additionally, the calculation of the limit of detection (LOD) is performed with the equation described below [43]:

(2) $$LOD=\frac{3\sigma }{slope}$$

where σ represents the standard deviation of the blank response value. The slope can be determined from the concentration–response curve. The LOD of the L_0.8_In_0.2_FO sensor is 133 ppb, lower than that of the LFO sensor (246 ppb). This result reveals that the appropriate amount of In^3+^ doping can improve the sensitivity of the LFO NFs and enable the efficient detection of trace HCHO (ppb level).

The sensor’s selectivity was evaluated on exposure to 100 ppm of HCHO, ethanol, methanol, acetone, toluene, and benzene, respectively, at 180 °C (Figure 5f). All the sensors show much higher response values to HCHO than other interfering gases, indicating that the sensors possess high selectivity to HCHO gas. In addition, the impact of the ambient relative humidity (RH) on the sensor’s performance is considerable in practical applications. An investigation has been conducted to assess the impact of the humidity levels, varying from 33% to 70% relative humidity (RH), on the L_0.8_In_0.2_FO sensor’s sensitivity to 100 ppm of HCHO. As displayed in Figure 5g, the response value of the L_0.8_In_0.2_FO sensor gradually decreases as the humidity increases because of the occupied active sites on the surface of the sensing material by water molecules [44]. Even at a high relative humidity of 70%, the L_0.8_In_0.2_FO sensor maintains a response level that is approximately 58% of its initial sensitivity.

Figure 5h presents the five cycles of the L_0.8_In_0.2_FO sensor toward 100 ppm HCHO at 180 °C. The observation reveals that the L_0.8_In_0.2_FO sensor exhibits a slight fluctuation in its response value and maintains consistent response and recovery times, which is indicative of its excellent reproducibility. Furthermore, over the 30 days of continuous monitoring, the L_0.8_In_0.2_FO sensor’s response to HCHO shows only a minor fluctuation of 10%, manifesting its good long-term stability. A comparison of the L_0.8_In_0.2_FO sensor versus other reported HCHO sensors is presented in Table S2. The sensor based on the L_0.8_In_0.2_FO NFs exhibits the best gas sensing characteristics among these sensors, operating at a low temperature with high sensitivity and boasting rapid response and recovery times, suggesting its promising application in the real-time detection of HCHO.

### 3.3. Gas Sensing Mechanism

The sensing mechanism of LaFeO_3_ can be interpreted through the alterations in its electrical resistance in response to the presence of the target gas. As a typical p-type semiconductor, LaFeO_3_ is characterized by the presence of holes (h) as the predominant charge carriers, which are generated by the ionization of La^3+^ cation vacancies [41]. The formation of charge carrier holes (h) can be represented using Kroger–Vink defect notations, as illustrated below [12]:

(3) $$V_{La}\to \;V_{La}^{\prime \prime \prime }+3\;h$$

**Figure 6:**
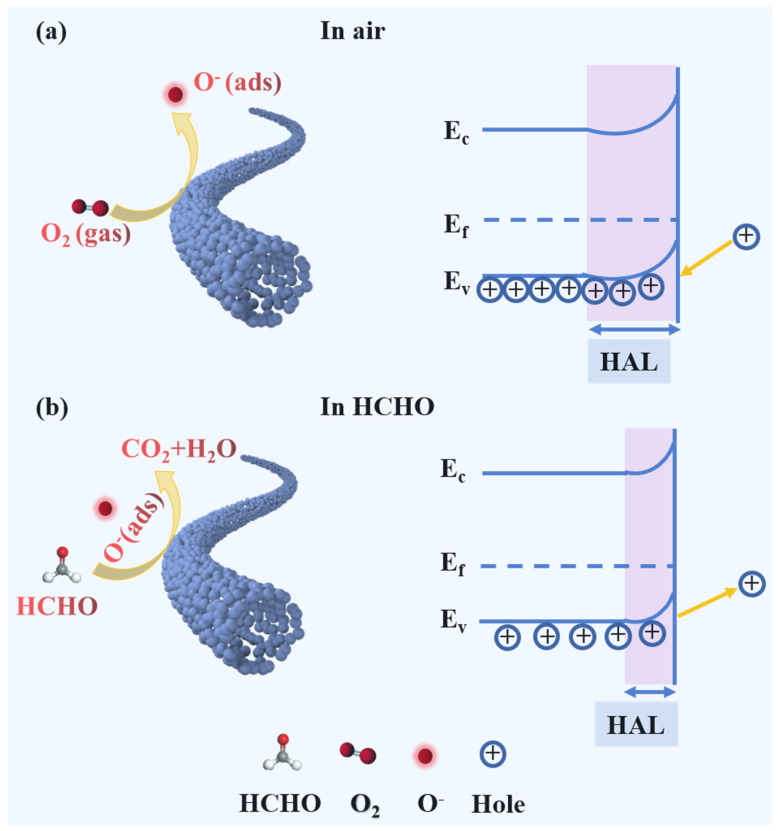
Schematic of the sensing mechanism and energy band of the L_0.8_In_0.2_FO NFs (**a**) in air and (**b**) in ambient HCHO.

As depicted in Figure 6, upon exposure to air, oxygen molecules adsorb onto the surface and capture electrons from the conduction band of LFO, leading to the creation of various species of chemisorbed oxygen [45]. The process can be described as below:

O_2_ (gas) → O_2_ (ads) (4)

O_2_ (ads) + e^−^ → O_2_^−^ (ads) (T < 100 °C) (5)

O_2_^−^ (ads) + e^−^ → 2O^−^ (ads) (100 °C < T < 300 °C) (6)

O^−^ (ads) + e^−^ → O^2−^ (ads) (T > 300 °C) (7)

The electron transfer processes will increase the hole and form a thick hole accumulation layer (HAL) at the surface of LaFeO_3_, reducing the resistance of the sensing material (Figure 6a) [46]. When the LaFeO_3_ sensor is exposed to the reducing gas of HCHO, the HCHO gas molecule will adsorb on the surface and react with the chemisorbed oxygen. The electrons captured by the adsorbed oxygen are released back to the valence band and recombined with the holes, resulting in the thinner HAL and increased resistance (Figure 6b). In this study, the optimal operating temperature of the gas sensor is 180 °C; thus, the oxygen species is O^−^. The corresponding reactions can be described as follows:

HCHO (gas) + 2O^−^ (ads) → CO_2_ (gas) + H_2_O (gas) + 2e^−^ (8)

e^−^ + h → null (9)

The enhanced HCHO gas sensing capabilities of the L_0.8_In_0.2_FO NFs can be attributed to the following facets:

(i) The enhanced oxygen vacancies generated by the doping of In^3+^ ions. Oxygen vacancies commonly serve as positive charge centers, and the electrons around oxygen vacancies are more susceptible to being captured by oxygen molecules (reactions (10) and (11)). The presence of a moderate number of oxygen vacancies is beneficial for the adsorption of oxygen onto the surface of the sensing material and enables the formation of more chemisorbed oxygen species [47]. As can be seen from the O 1s XPS spectra (Figure 4d and Figure S4) and Table S1, the proportion of the Ov component rises with the increase of In^3+^ doping and reaches the maximum value of 38.9% in the L_0.8_In_0.2_FO NFs. The reaction process can be described as follows:

(10) $$O_{o}^{X}\to \;\frac{1}{2}\;O_{2}+V_{o}^{\prime }+e^{-}$$

(11) $${\;V}_{o}^{\prime }\to \;V_{O}^{\prime \prime }+e^{-}$$

Furthermore, the oxygen vacancies in the L_0.8_In_0.2_FO NFs are demonstrated to reduce the bandgap width [48], as confirmed through the UV–vis DRS spectra in Figure 4e. A narrowed bandgap width results in a decreased energy requirement for electron transitions and concurrently accelerates the electron transfer process [49], improving the gas sensing performance.

(ii) The large surface area and porous structure. The BET analysis indicates that the L_0.8_In_0.2_FO NFs possess a larger specific surface area compared to the pristine LFO NFs, which increases the number of active adsorption sites and is conducive to the adsorption of oxygen and HCHO gas molecules [50]. In addition, the mesoporous structure of the L_0.8_In_0.2_FO NFs enhances the diffusive transport of the target gases into the material, thereby contributing to more effective interaction with the active sites on the sensor’s surface and achieving a faster response time and higher sensitivity.

## 4. Conclusions

In summary, various L_x_In_1-x_FO NFs with different ratios of La/In were synthesized by the electrospinning method and subsequent calcination route. The O_v_ concentration can be tuned by modulating the amount of indium doping. The gas sensing test proved that the doping of In^3+^ ions greatly enhances the HCHO sensing capabilities of the LFO. The results show that the gas sensor based on the L_0.8_In_0.2_FO NFs reached the highest response of 18.8 toward 100 ppm HCHO at the optimum operating temperature of 180 °C, which is higher than that of the pristine LFO NFs (4.2). Moreover, the fast response/recovery time (2 s/22 s), outstanding repeatability, and excellent long-term stability of the L_0.8_In_0.2_FO sensor were also demonstrated. Further investigations revealed that the improved HCHO sensing performance of the L_0.8_In_0.2_FO NFs can be attributed to the abundant oxygen vacancies generated by the In^3+^ quantitative replacement of La^3+^ in the A-site, along with the increased surface area and the porous structure of the L_0.8_In_0.2_FO NFs. This work highlights the potential of the sensor based on the L_0.8_In_0.2_FO NFs for the detection of HCHO in practical sensor applications.

## Data Availability

The data that have been used are confidential.

## Supplementary Materials

The following supporting information can be downloaded at: https://www.mdpi.com/article/10.3390/nano14191595/s1, Text S1: Gas sensor fabrication and sensing performance tests; Figure S1: Photographic images and schematic diagram of a fabricated gas sensor; Figure S2: N_2_ adsorption-desorption isotherms and pore-size distributions of (a) L_0.9_In_0.1_FO and (b) L_0.7_In_0.3_FO NFs; Figure S3: XPS survey spectra of four L_x_In_1-x_FO samples; Figure S4: XPS spectra of O 1s of (a) L_0.9_In_0.1_FO and (b) L_0.7_In_0.3_FO NFs; Table S1: The average crystallite size for L_x_In_1-x_FO NFs by considering the (121) crystal plane from their XRD patterns; Table S2: The relative percentages of three different oxygen species for four L_x_In_1-x_FO samples; Table S3: Comparison of HCHO gas sensing performance with other gas sensors. References [51,52,53,54,55] are cited in the supplementary materials.
