# Supplementary material for: Efficient Formaldehyde Gas Sensing Performance via Promotion of Oxygen Vacancy on In-Doped LaFeO3 Nanofibers
Source: Nanomaterials (Basel). 2024 Oct 2;14(19):1595. doi: 10.3390/nano14191595 (PMC11478380; doi:10.3390/nano14191595)
Supplement: Supplementary file 1 [file nanomaterials-14-01595-s001.zip › nanomaterials-3181424-supplementary.pdf]

Supplementary Materials

Efficient formaldehyde gas sensing performance by promoting oxygen vacancy on In-doped $LaFeO_3$ nanofibers

Lei Zhu [a,b], Jiaxin Zhang [a], Jianan Wang [a*], Jianwei Liu [a,c], Wei Zhao [b], Wei Yan [a*]

[a] Xi'an Key Laboratory of Solid Waste Resource Regeneration and Recycling, State Key Laboratory of Multiphase Flow Engineering, School of Energy and Power Engineering, Xi'an Jiaotong University, Xi'an 710049, China

[b] School of Physics and Electrical Engineering, Weinan Normal University, Chaoyang Street, Weinan 714099, China

[c] School of Chemistry and Chemical Engineering, Xi'an University of Science & Technology, Xi'an 710054, China

*** Corresponding author.**

E-mail address: wangjn116@xjtu.edu.cn (Jianan Wang);

yanwei@xjtu.edu.cn (Wei Yan).

Text S1: Gas sensor fabrication and sensing performance tests

As shown in Figure S1, an appropriate amount of $L_xIn_{1-x}FO$ sensing materials were blended with deionized water to form a consistent slurry. Subsequently, the paste was coated on the ceramic tube with Au electrodes by a small brush to form a uniform thick film. To enhance the stability, the as-prepared gas sensor was subjected to a process of aging at 100 ℃ for 7h. The ceramic tube consists of four sections including a ceramic tube, a Ni − Cr heater, Au electrodes and Pt wires. The heater is placed into the ceramic tube to provide the working temperature, and the comb-shaped Au electrodes are set outside the tube with the sensing materials coated on it. The sensing layer is coated on the ceramic tube to provide the gas signals.

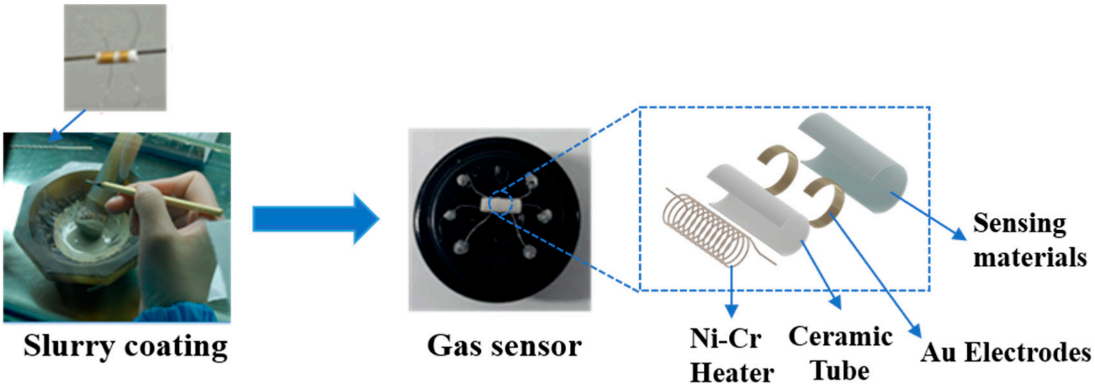

Figure S1. Photographic images and schematic diagram of a fabricated gas sensor.

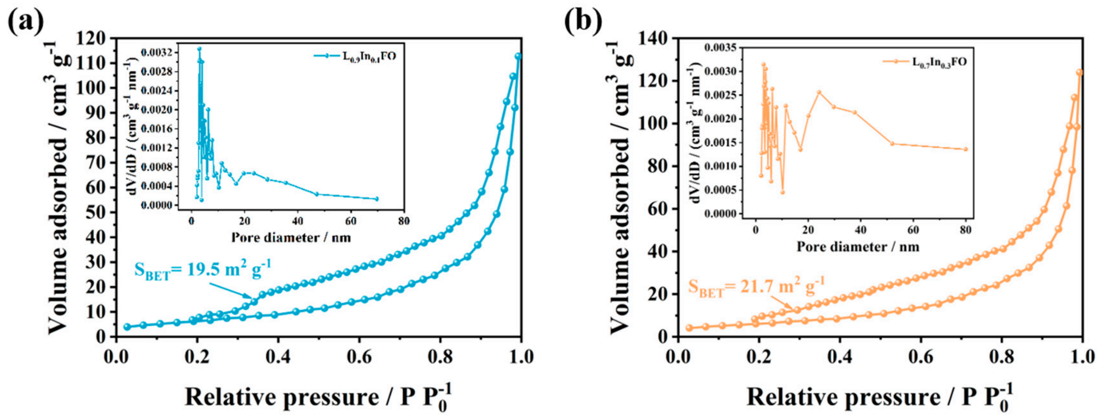

Figure S2. N_2 adsorption-desorption isotherms and pore-size distributions of (a) $L_{0.9}In_{0.1}FO$ and (b) $L_{0.7}In_{0.3}FO$ NFs.

As depicted in Figure S2, the calculated pore size of $L_{0.9}In_{0.1}FO$ and $L_{0.7}In_{0.3}FO$ NFs is mainly distributed about 2.9 and 3.1 nm, respectively.

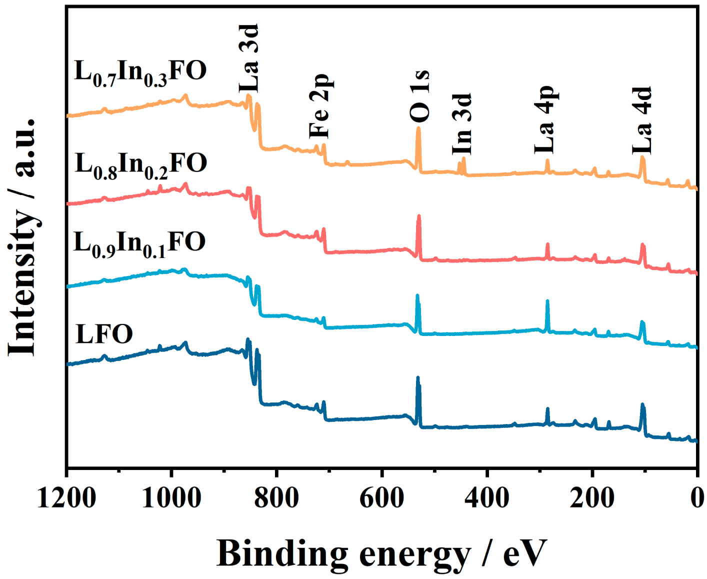

Figure S3. XPS survey spectra of four $L_xIn_{1-x}FO$ samples

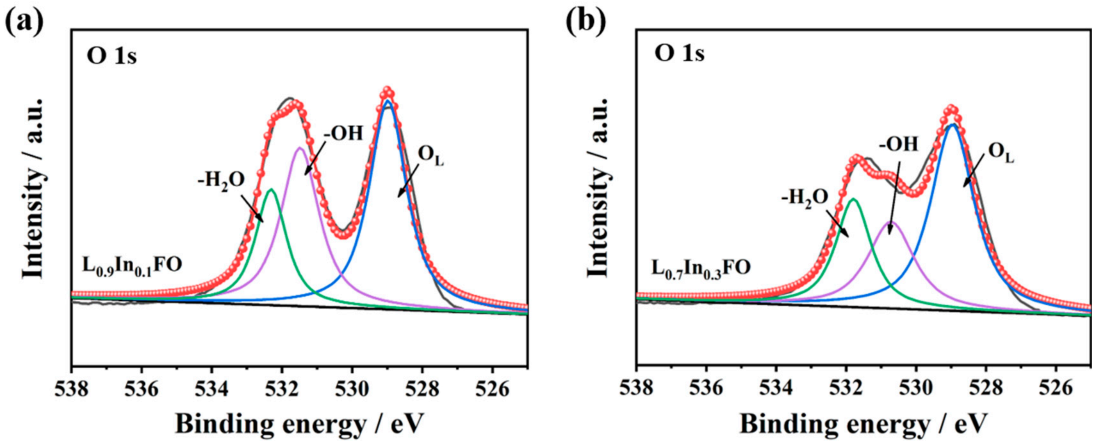

Figure S4 XPS spectra of O 1s of (a) $L_{0.9}In_{0.1}FO$ and (b) $L_{0.7}In_{0.3}FO$ NFs.

Table S1. The average crystallite size for $L_xIn_{1-x}FO$ NFs by considering the (121) crystal plane from their XRD patterns.

Materials	2Theta (degree)	FWHM (degree)	Crystallite size (nm)
LFO	32.22	0.437	18.9
$L_{0.9}In_{0.1}FO$	32.259	0.447	18.5
$L_{0.8}In_{0.2}FO$	32.28	0.47	17.5
$L_{0.7}In_{0.3}FO$	32.32	0.466	17.7

Table S2. The relative percentages of three different oxygen species for four $L_xIn_{1-x}FO$ samples

Sample	Oxygen Species	Relative Percentage (%)
LFO	O_L	51.7
	-OH	27.6
	H_2O	20.7
$L_{0.9}In_{0.1}FO$	O_L	44.7
	-OH	34.7
	H_2O	20.6
$L_{0.8}In_{0.2}FO$	O_L	37.6
	-OH	38.9
	H_2O	23.5
$L_{0.7}In_{0.3}FO$	O_L	50.8
	-OH	25.3
	H_2O	23.9

Table S3. Comparison of HCHO gas sensing performance with other gas sensors.

Materials	Temperature / °C	Concentration / ppm	Response	t_{res}/t_{rec} / s	Ref.
In_2O_3/Co_3O_4	180	100	15.7	32 / 42	[1]
Au-In_2O_3	240	100	37	3 / 8	[2]
SnO_2	200	100	38.3	17 / 25	[3]
SnO_2/Fe_2O_3	220	20	4.5	9 / 34	[4]
Er/In_2O_3	260	20	12	5 / 38	[5]
Ag-$LaFeO_3$	230	5	4.8	2/ 4	[6]
In-$LaFeO_3$	125	100	122	36/ 40	[7]
$L_{0.8}In_{0.2}FO$ NFs	180	100	18.8	2 / 22	**This work**

References

1. Cao, J.; Zhang, N.R.; Wang, S.M.; Zhang, H.M. Electronic structure-dependent formaldehyde gas sensing performance of the In_2O_3/Co_3O_4 core/shell hierarchical heterostructure sensors. *J. Colloid Interface Sci.* **2020**, *577*, 19-28, doi:10.1016/j.jcis.2020.05.028.
2. Zhang, S.; Song, P.; Li, J.; Zhang, J.; Yang, Z.; Wang, Q. Facile approach to prepare hierarchical Au-loaded In_2O_3 porous nanocubes and their enhanced sensing performance towards formaldehyde. *Sens. Actuators, B* **2017**, *241*, 1130-1138, doi:10.1016/j.snb.2016.10.023.
3. Li, Y.; Chen, N.; Deng, D.; Xing, X.; Xiao, X.; Wang, Y. Formaldehyde detection: SnO_2 microspheres for formaldehyde gas sensor with high sensitivity, fast response/recovery and good selectivity. *Sens. Actuators, B* **2017**, *238*, 264-273, doi:10.1016/j.snb.2016.07.051.
4. Lou, C.; Huang, Q.; Li, Z.; Lei, G.; Liu, X.; Zhang, J. Fe_2O_3-sensitized SnO_2 nanosheets via atomic layer deposition for sensitive formaldehyde detection. *Sens. Actuators, B* **2021**, *345*, doi:10.1016/j.snb.2021.130429.
5. Wang, X.S.; Zhang, J.B.; Wang, L.Y.; Li, S.C.; Liu, L.; Su, C.; Liu, L.L. High response gas sensors for formaldehyde based on Er-doped In_2O_3 nanotubes. *Journal of Materials Science & Technology* **2015**, *31*, 1175-1180, doi:10.1016/j.jmst.2015.11.002.
6. Wei, W.; Guo, S.; Chen, C.; Sun, L.; Chen, Y.; Guo, W.; Ruan, S. High sensitive and fast formaldehyde gas sensor based on Ag-doped $LaFeO_3$ nanofibers. *J. Alloys Compd.* **2017**, *695*, 1122-1127, doi:10.1016/j.jallcom.2016.10.238.
7. Xiao, C.; Zhang, X.; Ma, Z.; Yang, K.; Gao, X.; Wang, H.; Jia, L. Formaldehyde gas sensor with 1 ppb detection limit based on In-doped $LaFeO_3$ porous structure. *Sens. Actuators, B* **2022**, *371*, doi:10.1016/j.snb.2022.132558.
